# Gold Nanoparticles Surface Plasmon Resonance Enhanced Signal for the Detection of Small Molecules on Split-Aptamer Microarrays (Small Molecules Detection from Split-Aptamers)

**DOI:** 10.3390/microarrays4010041

**Published:** 2015-02-09

**Authors:** Feriel Melaine, Yoann Roupioz, Arnaud Buhot

**Affiliations:** 1 Univ. Grenoble Alpes, INAC-SPRAM, F-38000 Grenoble, France; E-Mails: melferiel87@hotmail.com (F.M.); yoann.roupioz@cea.fr (Y.R.); 2 CEA, INAC-SPrAM, F-38000 Grenoble, France; 3 CNRS, INAC-SPrAM, F-38000 Grenoble, France

**Keywords:** split-aptamers, Surface Plasmon Resonance imaging, gold nanoparticles, small molecules detection

## Abstract

The detection of small molecules by biosensors remains a challenge for diagnostics in many areas like pharmacology, environment or homeland security. The main difficulty comes from both the low molecular weight and low concentrations of most targets, which generally requires an indirect detection with an amplification or a sandwich procedure. In this study, we combine both strategies as the amplification of Surface Plasmon Resonance imaging (SPRi) signal is obtained by the use of gold nanoparticles and the sequence engineering of split-aptamers, short oligonucleotides strands with strong affinity towards small targets, allows for a sandwich structure. Combining those two strategies, we obtained state-of-the-art results in the limit of detection (LOD = 50 nM) with the model target adenosine. Furthermore, the SPRi detection led on aptamer microarrays paves the way for potential multi-target detections thanks to the multi-probe imaging approach.

## 1. Introduction

Small molecules detection is of paramount importance in many areas ranging from pharmacology to environmental diagnostic [1,2,3]. The main difficulty in such diagnostic relates to the small size and low concentrations of the encountered targets [4]. Direct detection is generally impossible as the effect of the binding of the targets to the probes is usually close to noise signal. Indirect strategies are to be found in order to detect and quantify small molecules. The folding of the probes upon binding of the target is one common strategy. To this end, aptamers, short oligonucleotides selected specifically with high affinity towards a predefined target, present such behavior [5,6]. The sensitivity and selectivity of the aptamer towards the target is principally due to a particular three-dimensional folding (hairpins, three‑way junctions or G-quadruplex for example) enabling the target binding [7,8]. In fact, their three dimensional structure is necessary for and may be induced by the binding of the target. Whereas their folding upon binding of the target is relevant for proteins like thrombin, for small molecules, the aptamer is generally pre-folded before the target binds [9]. However, in recent years, a limited class of aptamers have been shown to keep most of their affinity despite the splitting of the sequence in two parts [10,11,12,13,14,15]. This is particularly the case of the adenosine aptamer whose hairpin sequence may be cut in the loop as this region is not directly involved in the adenosine binding [16,17]. Due to the two strands binding the targets, sandwich assays are possible, thus leading to the indirect detection of the targets. Solution phase detection using split-aptamers for the detection of adenosine gave detection limit of few micromolars.

In this study, we would like to extend this approach to a microarray format with Surface Plasmon Resonance imaging (SPRi) detection although SPRi has been known so far to be limited in term of resolution for the direct detection of small molecules (Molecular weight below 1 kDa) [18,19,20]. While good performances have been obtained for the detection of proteins [18,21,22], indirect strategies or improved sensibility should be considered for small molecules [23]. As an example, it can be done by taking benefit of using gold nanostructures for the sensitive detection of targets by plasmonic effects like SERS [24,25,26]. To this end, introducing gold nanoparticles in the detection process is a common strategy used to amplify SPRi signal due to their dual effect on plasmon (the standard mass effect and a coupling of the localized surface plasmon of the particles with the SPRi signal) [27]. However, this strategy requires a sandwich assay where two probes are respectively grafted on the biosensor surface and on the nanoparticles [28]. Split-aptamers are thus ideal probe candidates. In this paper, we combine both strategies: SPRi amplification signal thanks to gold nanoparticles along with sequence engineering of split-aptamer for sandwich assay format of detection. We developed dedicated functionalization chemistries of both gold surfaces, the SPRi prisms and the nanoparticles, in order to limit the non-specific adsorption. We analyzed the limit of detection for two particular adenosine split‑aptamer sequences. Three orders of magnitude difference in the LODs were observed depending on the existence or not of reference signal in absence of adenosine. For the split-aptamer sequence presenting a signal OFF- signal ON behavior, state-of-the-art performances have been obtained with a LOD as low as 50 nM.

## 2. Experimental Section 

### 2.1. Reagents and Chemicals

Gold nanoparticles (AuNPs) with a diameter of 20 nm were purchased from BBI Solutions (Cardiff, UK). Adenosine, guanosine, HO-(CH_2_)_11_-PEG-SH (MW 336.5Da) named PEG300, Bis-(p-sulfonatophenyl) phenylphosphine dihydrate dipotassium (BSPP) and all buffer reagents were purchased from Sigma‑Aldrich (Saint Quentin Fallavier, France). CH_3_O-PEG-SH (MW 2000 Da) named PEG2000, was purchased from Rapp Polymere GmbH (Tübingen, Germany). Different concentrations of adenosine and 1 µM guanosine were prepared in the SPR running buffer (HEPES 10 mM, MgCl_2_ 5 mM, NaCl 150 mM and 0.005% Tween20, pH 7.4). Ultrapure water (18.2 MΩ·cm) was used throughout.

The oligonucleotides were purchased from Eurogentec (Angers, France) with a thiol modification at their 5' position and a thymine spacer (five or ten thymine nucleotide) before the sequence of interest. Sequences are listed in Table 1. APT4 and APT8 correspond to the full adenosine aptamers and Split‑APT4, Split‑APT8 and Split-APT are their split sequences, designed according to the literature [17]. CN8 sequence was used as a negative control.

**Table 1:** Oligonucleotide sequences.

Name	Sequence (5' > 3')
APT8	HS-T_5_-AGAGAACCTGGGGGAGTATTGCGGAGGAAGGTTCTC
APT4	HS-T_5_-AGAGAACCTGGGGGAGTATTGCGGAGGAAGGTAGAG
Split-APT8	HS-T_10_-TGCGGAGGAAGGTTCTC
Split-APT4	HS-T_10_-TGCGGAGGAAGGTAGAG
Split-APT	HS-T_10_-AGAGAACCTGGGGGAGTAT
CN8	H_2_N-T_5_-TAAGTTCATCTCCCCGGTGGTGGTTGTGGTT

### 2.2. DNA Microarray Fabrication

Thiolated oligonucleotides were grafted on gold-coated prisms (Horiba Scientific-GenOptics, Orsay, France) as Self-Assembled Monolayers (SAM) according to the protocol described in previous work [21,22]. Briefly, the gold surface lying on the prism was first cleaned by plasma treatment (0.6 mbar, 75% Oxygen, 25% Argon, power 40 W, 6 min) in a plasma generator (Femto, Diener Electronic, Ebhausen, Germany). Then, droplets of approximately 4 nL of HK_2_PO_4_ buffer (1 M, pH 9.25), containing a mixture of 20 µM of thiol-modified DNA probes and 10 µM of PEG2000 or PEG300 solution, were deposited on the surface using a piezoelectric dispensing system (Siliflow, Valence, France). The deposition was done under a controlled atmosphere of 85% humidity and left at room temperature for 60 min. After overnight drying, prisms were thoroughly rinsed with deionized water and dried under argon stream for few seconds. The size of the spots is approximately 500 µm in diameter, with a 1.3 mm pitch. The probes coverage surface is estimated to 8 pmol/cm² [22]. Prior to the SPR measurements, the functionalized prism surface is blocked with a PEG2000 or PEG300 solution (150 µM) for 60 min incubation at room temperature. Then, surfaces were rinsed with deionized water and dried under argon stream.

### 2.3. AuNPs-ssDNA Conjugation

Gold nanoparticles modified by ss-DNA were prepared according to the literature with some modifications [29]. In a typical experiment, to 1mL of citrate coated AuNPs solution (1.16 nM), 3 mg of BSPP was added, and the mixture was stirred at room temperature in order to allow phosphine ligands to replace the citrate ligands. Following overnight incubation, 2 mg of NaCl were added to the stirring mixture until the color change from red to cloudy purple. The solution was centrifuged at room temperature for 10 min at 16750 G to collect the precipitated AuNPs. The supernatant was removed and the pellet re-suspended in BSPP buffer (1 mg/mL of BSPP). Nanoparticle purification and concentration steps were carried out in a Sigma Laborzentrifugen 3K15 centrifuge.

To functionalize AuNPs with ssDNA conjugates, 10 µL of 5'-thiolated DNA at 10 µM (PBS, pH 7.4) was mixed with 70 µL of the concentrated gold colloid prepared in the first step, 10 µL of PBS (pH 7.4) and 10 µL of BSPP buffer (1 mg/20 µL of BSPP). The mixture was incubated under gentle agitation at room temperature for 24 h, then 7 µL of PEG solution at 150 µM (PBS, pH 7.4) was added and the solution was incubated with gentle agitation for a further 2 h. The solution was then centrifuged (10 min, 16750 G) to remove the excess of reagents. The supernatant was discarded and the precipitate was washed with the SPR running buffer. After a second centrifugation, the final precipitate was re‑suspended in 1 mL of SPR running buffer. UV-visible absorption spectroscopy was used for optical characterization of the AuNPs-ssDNA conjugates. The measurements were taken using a ND-1000 Nanodrop (Labtech International, Uckfield, UK) spectrometer. AuNPs and AuNPs-ssDNA conjugates were quantified by measuring the absorbance at λ = 520 nm and calculating the concentration using Beer’s law based on their extinction coefficients: ε_520_ (20 nm) = 8.8 × 10^8^ M^−1^·cm^−1^. The maximum UV–Vis absorption peak of unmodified AuNPs and AuNPs-ssDNA conjugates in solution were 520 nm and 528 nm, respectively.

### 2.4. SPRi Measurements

As for previous work [21,22], the SPRi experiments were carried out using a SPR imager apparatus (SPRi-Lab, Horiba Scientific-GenOptics) equipped with an incoherent light source (λ = 635 nm). The reaction chamber consists of a hexagonal reactor PEEK flow cell (~15 µL of volume). The flow cell was connected to PEEK tubing coupled with a degassing system (Alltech, Carquefou, France) and a syringe Cavro pump (Tecan, San Jose, CA, USA). The experiments were performed at 25 °C. All the injections were dispensed using a 500 µL injection loop. The SPR data were acquired using the software furnished by Horiba Scientific-GenOptics. Acquisition of the reflectivity signal, registered with a 12-bit camera, was launched upon stabilization of the baseline. The reflectivity values were averaged over the replicates of each spot series and plotted upon time.

For the hybridization experiments, each ssDNA injection (at 1 µM) or AuNPs-ssDNA conjugates injection (at 200 pM) was dispensed via a flow rate of 50µL/mL or an alternating back-and-forth flow mode (15 µL dispensed volume, 10 µL aspirated volume, 50 µL/min flow rate). For biochip recycling, 50 mM NaOH was injected for 8min in order to denature the hybridized complementary strands of DNA and regenerate single stranded DNA on control spots. Concerning the adenosine detection assays, a series of adenosine solutions (from 1 nM to 1 µM) were co-injected with AuNPs-SplitAPT conjugates (200 pM) under the alternating flow. To assess the target-specificity of the sensing system, 1 µM guanosine solution was also co-injected with AuNPs-SplitAPT conjugates (200 pM).

## 3. Results and Discussion

### 3.1. Functionalization of Gold Surfaces

In view of the detection of small molecules by SPRi detection, we aimed at an amplification of the SPRi signal by the use of gold nanoparticles (AuNPs). Those nanoparticles have two main effects: besides an amplification phenomenon due to their mass, we may also expect a coupling between the localized surface plasmon of the AuNPs and the surface plasmons of the biosensor itself [30]. The latter effect is present as soon as the AuNPs are sufficiently close to the sensor surface (distance lower than 10 nm) [31,32]. However, this amplification requires successful functionalization on both gold surfaces with anti-fouling molecules in order to avoid non-specific adsorption. 

**Figure 1:**
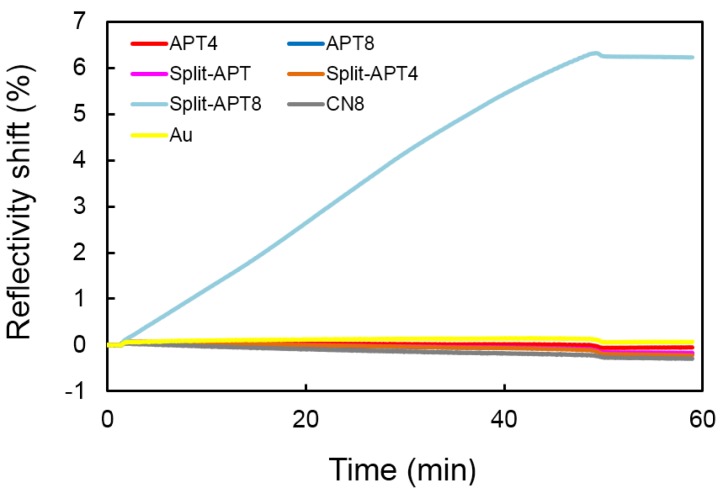
Reflectivity shifts observed upon injection of gold nanoparticles grafted with Split-APT sequences on various spots of the Surface Plasmon Resonance imaging (SPRi) biosensor surface without the target adenosine present in the solution. Signal increase is observed only on Split-APT8 spots through hybridization of the hairpin stems of the split‑aptamers. APT8 and APT4 spots do not present signal shift due to a folding of the complete aptamer on the surface while Split-APT4 sequences present stems (four bases) too short to hybridize with the Split-APT sequences grafted on the gold nanoparticles. Lack of non-specific signal is also confirmed on control spots with sequences CN8 and Split-APT or on pure gold.

PEG molecules are commonly used to decrease protein spontaneous adsorptions on surfaces and thus increase signal to noise ratios. This functionalization approach was successfully used for the detection of proteins by aptamer microarrays [21,22]. We adapted this method to the case of sandwich assays with split-aptamers grafted to the gold surface of SPRi prisms and nanoparticles. Two different lengths of thiolated PEG molecules have been tested for self-assembling monolayer (SAM) formation on gold: short and long ones of respectively 300 Da (PEG300) and 2 kDa (PEG2000). As can be seen from Figure 1, the longer PEG co-grafted with the split-aptamer sequences gave impressive results. While injecting gold nanoparticles grafted with Split-APT and PEG2000, no signal was observed on the gold surface of the SPRi prisms (Au curve in yellow). Furthermore, no signal was observed also on spots grafted with negative control sequences CN8 and PEG2000 (CN8 curve in grey). The lack of non‑specific interactions and SPRi signal on both cases insures that any SPRi signal observed on other spots effectively corresponds to specific interactions. 

The same experiments were realized with PEG300 on one or both gold surfaces (prisms or AuNPs). These trials were always leading to increased non-specific signals compared to PEG2000 grafting. Thus, the grafting with PEG300 was not considered for the following experiments, only PEG2000 co‑grafting of the spotsand the AuNPs were considered. In conclusion, it seems that the hydrophilic behavior brought by the long PEG chains avoid any non-specific interactions and justifies the use of this original grafting strategy for sandwich assays with AuNP SPRi signal amplification. 

### 3.2. Hybridization of Split-APT AuNPs without Adenosine 

**Figure 2:**
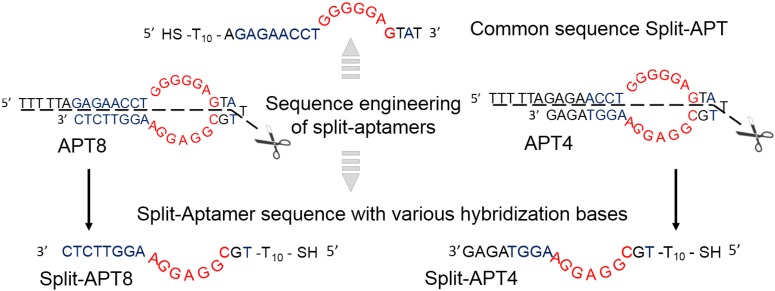
Aptamer sequence engineering into split-aptamers. Split-APT corresponds to the common part of both APT4 and APT8 split sequences whereas Split-APT4 and Split-APT8 are their respective counterparts.

The main advantage of SPRi detection compared to solution phase assays is the possibility to analyze multiple probes at the same time thanks to the microarray format. We used this opportunity to test various detection strategies and more particularly different aptamer sequences. First of all, two complete adenosine aptamers were considered and grafted to the microarrays. APT4 and APT8 only differ in the number of bases hybridizing in the stem domain close to the recognition site of the adenosine target. This variation of hybridizing bases affects the folding thermodynamics of the apamer and potentially its recognition affinity towards the targets. Besides those complete aptamers, the two corresponding split-aptamers were considered. Split-APT4 and Split-APT8 respectively correspond to the first part of the splitting of APT4 and APT8 whereas Split-APT is the second part common to both APT4 and APT8 (see Figure 2 for the sequence engineering). This last sequence was grafted to the AuNPs and used as a reference due to its possible interactions (in presence or not of adenosine) with the four considered sequences (APT4, APT8, Split-APT4 and Split-APT8). As a control sequence Split-APT was also grafted on the microarrays. A negative control CN8, completely independent from the adenosine aptamer was also considered on the microarrays. 

**Figure 3:**
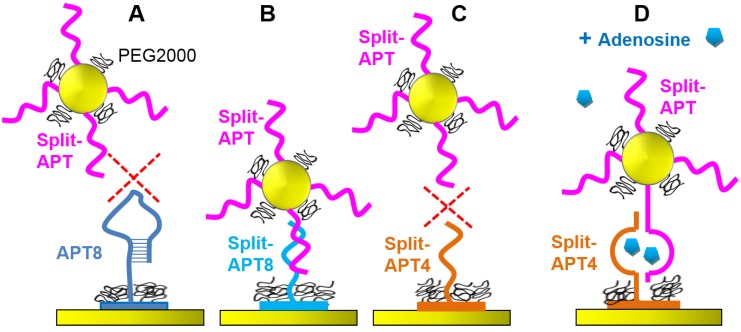
Split-APT gold nanoparticles interacting modes with the aptamer microarray: (**A**) Split-APT gold nanoparticles do not interact with APT8 spots due to the folding of the complete aptamer; (**B**) Split-APT gold nanoparticles interacts with Split-APT8 spots through hybridization even without adenosine; (**C**) Split-APT gold nanoparticles do not interact with Split-APT4 spots without adenosine, but (**D**) interacts with Split-APT4 spots in presence of adenosine.

First of all, we tested the interactions of the AuNPs grafted with Split-APT sequences on the microarray (Figure 1). Only spots grafted with Split-APT8 presented a shift in SPR reflectivity upon injection of 200 pM of AuNPs for 45 min at room temperature. This indicates that the eight complementary bases between Split-APT and Split-APT8 are sufficient to lead to specific hybridization (Figure 3B). Strangely enough, APT8, which also presents those eight bases complementary to Split‑APT, does not yielded specific signal (Figure 3A). This may be explained by the folded structure of APT8 at room temperature even without the presence of the target adenosine. This also confirms the difficulty to drive the folding of the aptamer by the presence of small targets since the folding is already present without the target. Finally, the spots Split-APT4 do not present specific signal suggesting that the four complementary bases do not hybridize at room temperature (Figure 3C). As we will see in the following sections, the presence of the adenosine targets stabilizes the complex formed between the split-aptamers sequences and allows for a specific signal on Split-APT4 spots (Figure 3D).

### 3.3. Adenosine Detection with Split-APT8 Sequence 

**Figure 4:**
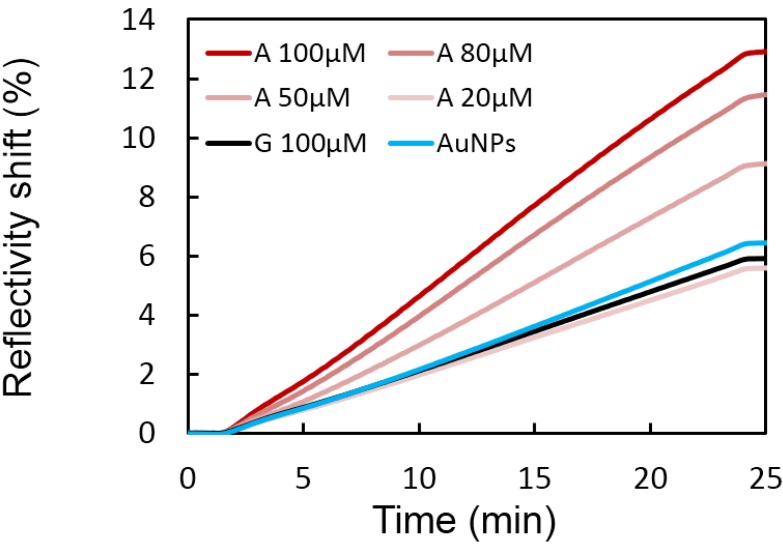
A series of different adenosine (A) concentrations have been incubated in presence of Split-APT coated gold nanoparticles. Higher SPRi signals on Split-APT8 spots are observed upon increased concentrations allowing a detection limit of 50 μM. The injection of 100 μM of guanosine (G) does not significantly modify the SPRi signal compared to the injection of gold nanoparticles (AuNPs) alone implying a good selectivity of the biosensor.

In this section, the detection limit of the sandwich assay with the APT8 splitting sequences were explored. AuNPs were grafted with Split-APT and the biosensor with Split-APT8 sequence. We have already seen that a specific signal (signal ON) is present without adenosine. A series of different adenosine concentrations ranging from 20 to 100 μM in presence of 200 pM of AuNPs were injected on the microarray in the same conditions as the injection of AuNPs alone. The injection of AuNPs was stopped after 24 min without reaching equilibrium. All the experiments present a leveling-off after the arrest of nanoparticles injection. The stability observed for the complexes between AuNPs functionalized with Split-APT and the Split-APT8 probe surface is high enough to avoid any desorption of the AuNPs. The stabilization of the complex formed by the target and the split-aptamers leads to an increased SPRi reflectivity shift for an increasing concentration of adenosine (Figure 4). A detection limit of 50 μM may be deduced from the range of adenosine concentration considered. The selectivity of the biosensor assay was validated by an injection of 100 μM of guanosine (G curve) whose reflectivity shift is comparable to the injection of AuNPs alone.

### 3.4. Adenosine Detection with Split-APT4 Sequence

**Figure 5:**
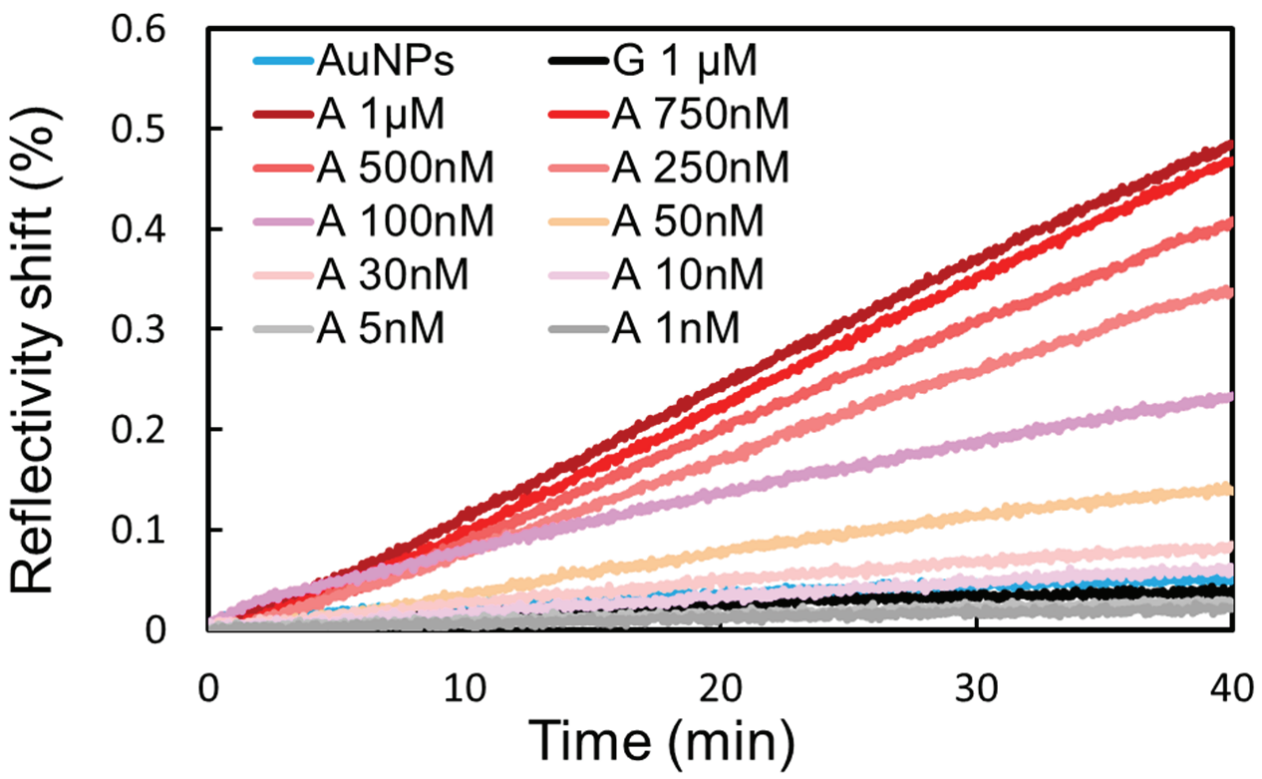
A range of adenosine (A) concentrations have been incubated in presence of Split-APT coated gold nanoparticles. Higher SPRi signals on Split-APT4 spots are observed upon increased concentrations allowing a detection limit of 50 nM. The injection of 1 μM of guanosine (G) does not significantly modify the SPRi signal compared to the injection of gold nanoparticles (AuNPs) alone implying a good selectivity of the biosensor.

The case of the biosensor grafted with Split-APT4 sequence differs from the previous one with Split-APT8 since no specific signal (signal OFF) is observed without adenosine. We injected a series of different adenosine solutions (from 1 nM to 1 μM) in presence of 200 pM of AuNPs in the same conditions than the injection of AuNPs alone. The injections of AuNPs with or without adenosine were longer and stopped after 40 min due to the reduced SPR signal observed compared to the Split-APT8 probes. The stabilization of the complex formed by the target and the split-aptamers leads to a specific signal (signal ON) with an increased SPRi reflectivity shift. This increased signal is dependent of the concentration of adenosine (Figure 5) with a limit of detection (LOD) of 50 nM. This LOD is three order of magnitude lower than the one obtained with Split-APT8 biosensor. Two main reasons may explain this huge effect. First of all, it is known that signal OFF—signal ON biosensors are generally more efficient than the ones with an increased signal ON in presence of the targets. This is principally due to the fact that it is easier to detect an increased signal form the background noise than from an important reference signal. However, this requires a limited non-specific signal to reduce the noise which is precisely the case with our grafting procedure. Furthermore, the sequence of the split‑aptamers may also explain a large part of the LOD improvement. Since the number of hybridization bases in the stem is reduced for Split-APT4 than for Split-APT8 the stabilizing effect of the target on the complex formation is relatively more important. Finally, the selectivity of the biosensor assay was also validated by an injection of 1 μM of guanosine (G curve) whose reflectivity shift is weak and comparable to the injection of AuNPs alone.

## 4. Conclusions

In this paper, we have described the detection of a small molecule, the adenosine, from a sandwich assay with split-aptamers based on a biosensor/microarray with SPRi detection. The combination of signal amplification and sequence engineering allowed us to obtained state-of-the-art performances for small molecules detection by SPR (LOD = 50 nM). Those results have been possible due to an efficient grafting strategy of both gold surfaces (SPRi biosensor prisms and gold nanoparticles). The grafting of long PEG molecules along with the oligonucleotide probes reduces the non-specific adsorption and significantly decreased the background noise. Furthermore, the use of gold nanoparticles grafted with a common Split-APT sequence allowed us not only to amplify the SPRi signal but also to test various aptamer probe sequences on the microarrays (APT4, APT8, Split-APT4 and Split-APT8). The Split-APT4 sequence gave better results than Split-APT8 (three orders of magnitude difference in LOD) whereas APT4 and APT8 sequences were not suitable to detect adenosine in sandwich assay with Split-APT grafted AuNPs. Although some cooperative effects may occur due to a large number of Split-APT sequences present on each AuNPs, the sequence engineering for the Split-APT4 and Split-APT8 enables to avoid background signal in absence of adenosine for the former sequence. The good performance of Split-APT4 probe is explained in part by the signal OFF—signal ON behavior of the biosensor. 

Furthermore, it is interesting to notice that the LOD obtained with this sandwich strategy is also two orders of magnitude lower than the dissociation constant of the binding of adenosine with the complete aptamer APT4 (K_D_ = 6 μM) [33,34]. AuNPs amplification of SPRi signal alone may not explain this impressive result. In fact, the size of the AuNP, 20 nm, and the number of Split-APT oligonucleotides per nanoparticles (few thousands) may lead to multiple interactions between Split-APT sequences on the same AuNP and Split-APT4 probes on the biosensor surfaces. The improved LOD is then related to the resulting cooperative behavior.

In conclusion, we have proven the interest of Surface Plasmon Resonance imaging for small molecules detection in a sandwich assay with split-aptamers. The microarrays format should allow us to generalize this approach to the multiple detection of small molecules in parallel.
